# Supplementary material for: Patients’ and Healthcare Professionals’ Experiences and Views of Recurrent Urinary Tract Infections in Women: Qualitative Evidence Synthesis and Meta-Ethnography
Source: Antibiotics (Basel). 2023 Feb 22;12(3):434. doi: 10.3390/antibiotics12030434 (PMC10044648; doi:10.3390/antibiotics12030434)
Supplement: Supplementary file 1 [file antibiotics-12-00434-s001.zip › antibiotics-2213021-supplementary.pdf]

Patients' and Healthcare Professionals' Experiences and Views of Recurrent Urinary Tract Infections in Women: Qualitative Evidence Synthesis and Meta-Ethnography

Supplementary material

Supplementary table S1. eMERGe (Improving reporting of Meta-Ethnography) reporting guidance checklist [21].

Criteria number	Criteria heading	Reporting Criteria	Location where item reported
**PHASE 1—SELECTING META-ETHNOGRAPHY AND GETTING STARTED**			
Introduction			
1	Rationale and context for the meta-ethnography	Describe the gap in research or knowledge to be filled by the meta-ethnography, and the wider context of the meta-ethnography	Page 2
2	Aim(s) of the meta-ethnography	Describe the meta-ethnography aim(s)	Page 2
3	Focus of the meta-ethnography	Describe the meta-ethnography review question(s) (or objectives)	Page 2
4	Rationale for using meta-ethnography	Explain why meta-ethnography was considered the most appropriate qualitative synthesis methodology	Page 2
**PHASE 2—DECIDING WHAT IS RELEVANT**			
Methods			
5	Search strategy	Describe the rationale for the literature search strategy	Pages 2-4
6	Search processes	Describe how the literature searching was carried out and by whom	Pages 2-4
7	Selecting primary studies	Describe the process of study screening and selection, and who was involved	Pages 4-5
Findings			
8	Outcome of study selection	Describe the results of study searches and screening	Figure 1 and pages 5-6
**PHASE 3—READING INCLUDED STUDIES**			
Methods			
9	Reading and data extraction approach	Describe the reading and data extraction method and processes	Pages 4-5
Findings			
10	Presenting characteristics of included studies	Describe characteristics of the included Studies	Table 2

Criteria number	Criteria heading	Reporting Criteria	Location where item reported
**PHASE 4—DETERMINING HOW STUDIES ARE RELATED**			
Methods			
11	Process for determining how studies are related	Describe the methods and processes for determining how the included studies are related:<br>- Which aspects of studies were compared<br>AND<br>- How the studies were compared	Pages 4-5
Findings			
12	Outcome of relating studies	Describe how studies relate to each other	Pages 4-5, tables 4 and 5
**PHASE 5—TRANSLATING STUDIES INTO ONE ANOTHER**			
Methods			
13	Process of translating studies	Describe the methods of translation:<br>- Describe steps taken to preserve the context and meaning of the relationships between concepts within and across studies<br>- Describe how the reciprocal and refutational translations were conducted<br>- Describe how potential alternative interpretations or explanations were considered in the translations	Pages 4-5
Findings			
14	Outcome of translation	Describe the interpretive findings of the translation	Pages 13- 28
**PHASE 6—SYNTHESIZING TRANSLATIONS**			
Methods			
15	Synthesis process	Describe the methods used to develop overarching concepts ("synthesised translations") Describe how potential alternative interpretations or explanations were considered in the synthesis	Page 5
Findings			
16	Outcome of synthesis process	Describe the new theory, conceptual framework, model, configuration, or interpretation of data developed from the synthesis	Page 28, Figures 2 and 3.
**PHASE 7—EXPRESSING THE SYNTHESIS**			
Discussion			
17	Summary of findings	Summarize the main interpretive findings of the translation and synthesis and compare them to existing literature	Pages 31-32

Criteria number	Criteria heading	Reporting Criteria	Location where item reported
18	Strengths, limitations, and<br>reflexivity	Reflect on and describe the strengths and limitations of the synthesis:<br>- Methodological aspects—for example, describe how the synthesis findings were influenced by the nature of the included studies and how the meta-ethnography was conducted.<br>- Reflexivity—for example, the impact of the research team on the synthesis findings	Page 5 and Page 32
19	Recommendations and conclusions	Describe the implications of the synthesis	Pages 32-33

Supplementary table S2. Characteristics of excluded studies table (ordered by study first author).

**Study ID**	**Reason for exclusion**
Ahmed 2016	Study design: This study did not use qualitative methods for both data collection and analysis
Al-Demour 2018	Study design: This study did not use qualitative methods for both data collection and analysis
Alraek 2001	Study design: This study did not use qualitative methods for both data collection and analysis (after review team discussion)
Asiimwe 2021	Topic of interest: This study did not include the relevant health issue, recurrent UTIs
Aspevall 2001	Topic of interest: This study did not include the relevant health issue, recurrent UTIs
Audulv 2013	Topic of interest: This study did not include the relevant health issue, recurrent UTIs
Bjorkman 2013	Topic of interest: This study did not include the relevant health issue, recurrent UTIs
Bjornsdottir 1996, 1998	Study design: This study did not use qualitative methods for both data collection and analysis
Bjornsdottir 2003, 2010	Topic of interest: This study did not include the relevant health issue, recurrent UTIs
Booth 2013	Topic of interest: This study did not include the relevant health issue, recurrent UTIs
Braund 2016	Topic of interest: This study did not include the relevant health issue, recurrent UTIs
Brookes-Howell 2018	Topic of interest: This study did not include the relevant health issue, recurrent UTIs
Coyne 2010	Topic of interest: This study did not include the relevant health issue, recurrent UTIs
Croghan 2019	Study design: this study was a conference abstract that relates to a full study that is included
Cutajar 2019	Conference abstract, PhD study currently not completed (correspondence from author)
Duane 2016	Topic of interest: This study did not include the relevant health issue, recurrent UTIs
Flottorp 2000	Study design: This study did not use qualitative methods for both data collection and analysis
Flottorp 2003	Topic of interest: This study did not include the relevant health issue, recurrent UTIs
Flower 2016a and b	Study design: this study was a conference abstract that relates to a full study that is included
Gbinigie 2019	Topic of interest: This study did not include the relevant health issue, recurrent UTIs
Gbinigie 2022	Topic of interest: This study did not include the relevant health issue, recurrent UTIs
Glogowska 2022	Topic of interest: This study did not include the relevant health issue, recurrent UTIs
Hartman 2021	Topic of interest: This study did not include the relevant health issue, recurrent UTIs
Hayes 2021	Topic of interest: This study did not include the relevant health issue, recurrent UTIs
Hosoglu 2021	Topic of interest: This study did not include the relevant health issue, recurrent UTIs
Jones 2020	Topic of interest: This study did not include the relevant health issue, recurrent UTIs
Jones 2021	Topic of interest: This study did not include the relevant health issue, recurrent UTIs
Jones 2021	Topic of interest: This study did not include the relevant health issue, recurrent UTIs

Study ID	Reason for exclusion
Langford 2019	Topic of interest: This study did not include the relevant health issue, recurrent UTIs
Lecky 2020	Topic of interest: This study did not include the relevant health issue, recurrent UTIs
Leile-van der Zande 2020	Study design: This study did not use qualitative methods for both data collection and analysis
Leydon 2009	Topic of interest: This study did not include the relevant health issue, recurrent UTIs
Leydon 2010	Topic of interest: This study did not include the relevant health issue, recurrent UTIs
Little 2009	Topic of interest: This study did not include the relevant health issue, recurrent UTIs
Lugtenberg 2010	Topic of interest: This study did not include the relevant health issue, recurrent UTIs
Malterud 1997	Topic of interest: This study did not include the relevant health issue, recurrent UTIs
Montorsi 2016	Study design: This study did not use qualitative methods for both data collection and analysis
Norris 2013	Topic of interest: This study did not include the relevant health issue, recurrent UTIs
Peiffer-Smadja 2020	Study design: This study did not use qualitative methods for both data collection and analysis (the analysis of service users)
Petruschke 2022	Topic of interest: This study did not include the relevant health issue, recurrent UTIs
Pill 1987	Topic of interest: This study did not include the relevant health issue, recurrent UTIs
Potter 2019	Topic of interest: This study did not include the relevant health issue, recurrent UTIs
Schweizer 2005	Topic of interest: This study did not include the relevant health issue, recurrent UTIs
Scott 2019a-d	Study design: this study was a conference abstract that relates to a full study that is included
Valmadrid 2021	Topic of interest: This study did not include the relevant health issue, recurrent UTIs
Vincent 2021	Topic of interest: This study did not include the relevant health issue, recurrent UTIs
Zektser 2019	Study design: this study was a conference abstract that relates to a full study that is included

Supplementary table S3. GRADE- CERQual (Confidence in the Evidence from Reviews of Qualitative research) Evidence profile. Table was developed using the GRADE-CERQual (iSoQ) online tool [30].

#	Summarised review finding	Methodological limitations	Coherence	Adequacy	Relevance	GRADE-CERQual assessment of confidence	References
**PATIENTS WITH RECURRENT UTIs**							

#	Summarised review finding	Methodological limitations	Coherence	Adequacy	Relevance	GRADE-CERQual assessment of confidence	References
**1**	Most women describe a long history of rUTI with variability of frequency, severity, symptoms, and ability to self-diagnose.	Minor concerns<br><br>**Explanation:** Minor concerns regarding methodological limitations: two studies of high quality, three of moderate quality and one study of low (lack of justification and detail on study design, no reflexivity and unclear recruitment) The study of low quality contributed minimally to the review theme.	No/Very minor concerns<br><br>**Explanation:** No/ very minor concerns regarding coherence: the review finding reflects the variability and complexity of the data from the primary studies.	Minor concerns<br><br>**Explanation:** Minor concerns regarding adequacy: four studies offering rich data and two offering thin data.	Minor concerns<br><br>**Explanation:** Minor concerns regarding relevance: four studies were directly relevant and two studies were partially relevant to the review question in terms of age of participants included and the potential for inclusion of data related to patients acute UTIs.	Moderate confidence<br><br>**Explanation:** Minor concerns regarding methodological limitations, No/Very minor concerns regarding coherence, Minor concerns regarding adequacy, and Minor concerns regarding relevance	Grigoryan et al. 2022 [35]; Croghan et al. 2021 [33]; Scott et al. 2021 [24]; Eriksson et al. 2014 [34]; Flower et al. 2014 [5]; Larcombe 2012 [38]
**2**	Various factors are described as triggers for rUTI, and patients worry about a serious underlying cause	Moderate concerns	No/Very minor concerns	Minor concerns	Minor concerns	Moderate confidence	Grigoryan et al. 2022 [35]; Groen and Lagro-Janssen 2005 [36]; Pat et al. 2020 [23];

#	Summarised review finding	Methodological limitations	Coherence	Adequacy	Relevance	GRADE-CERQual assessment of confidence	References
		**Explanation:** Moderate concerns regarding methodological limitations: one study of high quality, three of moderate quality and two studies of low quality (lack of justification and detail on study design and data collection methods, no reflexivity and unclear recruitment, possible selection bias and unclear ethical considerations)	**Explanation:** No/ very minor concerns regarding coherence: the review finding reflects the variability and complexity of the data from the primary studies.	**Explanation:** Minor concerns regarding adequacy: three studies offering rich data and three offering thin data.	**Explanation:** Minor concerns regarding relevance: two studies were directly relevant and four studies were partially relevant to the review question in terms of age of participants included and the potential for inclusion of data related to patients acute UTIs.	**Explanation:** Moderate concerns regarding methodological limitations, No/Very minor concerns regarding coherence, Minor concerns regarding adequacy, and Minor concerns regarding relevance	Gon-zalez et al. 2022 [31]; Flower et al. 2014 [5]; Larcombe 2012 [38]
**3**	Recurrent UTIs generally have significant and widespread effects on women's quality-of-life.	Moderate concerns<br><br>**Explanation:** Moderate concerns regarding methodological	No/Very minor concerns<br><br>**Explanation:** No/ very minor	Minor concerns<br><br>**Explanation:** Minor concerns regarding	Minor concerns<br><br>**Explanation:** Minor concerns regarding	Moderate confidence<br><br>**Explanation:** Moderate concerns regarding	Grigoryan et al. 2022 [35]; Groen and Lagro-Janssen 2005 [36]; Croghan et al. 2021 [33]; Pat et al. 2020 [23]; Gonzalez et al.

#	Summarised review finding	Methodological limitations	Coherence	Adequacy	Relevance	GRADE-CERQual assessment of confidence	References
		limitations: two studies of high quality, three of moderate quality and three studies of low quality (lack of justification and detail on study design and data collection methods, no reflexivity and unclear recruitment, possible selection bias and unclear ethical considerations). One of the low-quality studies contributed minimally to the review theme.	concerns regarding coherence: the review finding reflects the variability and complexity of the data from the primary studies.	adequacy: four studies offering rich data and four offering thin data.	relevance: four studies were directly relevant and four studies were partially relevant to the review question in terms of age of participants included and the potential for inclusion of data related to patients acute UTIs.	methodological limitations, No/Very minor concerns regarding coherence, Minor concerns regarding adequacy, and Minor concerns regarding relevance	2022 [31]; Eriksson et al. 2014 [34]; Flower et al. 2014 [5]; Larcombe 2012 [38]
**4**	Women with rUTI commonly use self-help, lifestyle, and Complementary and Alternative Medicines (CAM) options to try and treat and prevent UTIs.	Moderate concerns<br><br>**Explanation:** Moderate concerns regarding	No/Very minor concerns<br><br>**Explanation:** No/ very	Minor concerns<br><br>**Explanation:** Minor concerns	Minor concerns<br><br>**Explanation:** Minor concerns	Moderate confidence<br><br>**Explanation:** Moderate concerns	Grigoryan et al. 2022 [35]; Groen and Lagro-Janssen 2005 [36]; Pat et al. 2020 [23]; Scott et al. 2021 [24]; Gonzalez

#	Summarised review finding	Methodological limitations	Coherence	Adequacy	Relevance	GRADE-CERQual assessment of confidence	References
		methodological limitations: two studies of high quality, four of moderate quality and two studies of low quality (lack of justification and detail on study design and data collection methods, limited information on data analysis, no reflexivity and unclear recruitment, possible selection bias and unclear ethical considerations)	minor concerns regarding coherence: the review finding reflects the majority of the variability and complexity of the data from the primary studies.	regarding adequacy: five studies offering rich data and three offering thin data.	regarding relevance: four studies were directly relevant and four studies were partially relevant to the review question in terms of age of participants included and the potential for inclusion of data related to patients acute UTIs.	regarding methodological limitations, No/Very minor concerns regarding coherence, Minor concerns regarding adequacy, and Minor concerns regarding relevance	et al. 2022 [31]; Eriksson et al. 2014 [34]; Flower et al. 2014 [5]; Larcombe 2012 [38]
**5**	The effectiveness of antibiotics varied, and patients are concerned about their use.	Minor concerns<br><br>**Explanation:** Minor concerns regarding methodological limitations: one study of high quality, four studies	No/Very minor concerns<br><br>**Explanation:** No/ very minor concerns	Minor concerns<br><br>**Explanation:** Minor concerns regarding adequacy: four	Minor concerns<br><br>**Explanation:** Minor concerns regarding relevance:	Moderate confidence<br><br>**Explanation:** Minor concerns regarding methodological limitations,	Grigoryan et al. 2022 [35]; Gulliford et al. 2021 [37]; Pat et al. 2020 [23]; Scott et al. 2021 [24]; Gonzalez et al. 2022 [31]; Flower et al. 2014 [5];

#	Summarised review finding	Methodological limitations	Coherence	Adequacy	Relevance	GRADE-CERQual assessment of confidence	References
		of moderate quality and one study of low quality (lack of justification and detail on study design and data collection methods, no reflexivity and unclear recruitment, possible selection bias and unclear ethical considerations). The study of low quality had contributed minimally to the review theme	regarding coherence: the review finding reflects the variability and complexity of the data from the primary studies.	studies offering rich data and three offering thin data.	three studies were directly relevant and four studies were partially relevant to the review question in terms of age of participants included and the potential for inclusion of data related to patients acute UTIs.	No/Very minor concerns regarding coherence, Minor concerns regarding adequacy, and Minor concerns regarding relevance	Larcombe 2012 [38]
**6**	Women with rUTI seek healthcare for most, but not all, UTIs. They describe anger and frustration with healthcare in terms of their care, the use of antibiotics and an underestimation of the impact of rUTI.	Moderate concerns<br><br>**Explanation:** Moderate concerns regarding methodological limitations: two studies of high	Minor concerns<br><br>**Explanation:** Minor concerns regarding coherence: the review finding	No/Very minor concerns<br><br>**Explanation:** Very minor concerns regarding adequacy: five	Minor concerns<br><br>**Explanation:** Minor concerns regarding relevance: four studies were	Moderate confidence<br><br>**Explanation:** Moderate concerns regarding methodological limitations,	Grigoryan et al. 2022 [35]; Pat et al. 2020 [23]; Scott et al. 2021 [24]; Gonzalez et al. 2022 [31]; Eriksson et al. 2014 [34]; Flower et al. 2014 [5];

#	Summarised review finding	Methodological limitations	Coherence	Adequacy	Relevance	GRADE-CERQual assessment of confidence	References
		quality, four of moderate quality and one study of low quality (lack of justification and detail on study design and data collection methods, no reflexivity and unclear recruitment, possible selection bias and unclear ethical considerations)	reflects most of the variability and complexity of the data from the primary studies.	studies offering rich data and two offering thin data.	directly relevant and three studies were partially relevant to the review question in terms of age of participants included and the potential for inclusion of data related to patients acute UTIs.	Minor concerns regarding coherence, No/Very minor concerns regarding adequacy, and Minor concerns regarding relevance	Larcombe 2012 [38]
**7**	Women sought information and support from a variety of sources and feel more information and research is needed.	Moderate concerns<br><br>**Explanation:** Moderate concerns regarding methodological limitations: one study of high quality, three of moderate quality and one study of low quality (lack of	No/Very minor concerns<br><br>**Explanation:** No/ very minor concerns regarding coherence: the review finding reflects the variability and	No/Very minor concerns<br><br>**Explanation:** Very minor concerns: four studies offering rich data and one offering moderately rich data.	Minor concerns<br><br>**Explanation:** Minor concerns regarding relevance: three studies were directly relevant and two studies were partially	Moderate confidence<br><br>**Explanation:** Moderate concerns regarding methodological limitations, No/Very minor concerns regarding coherence,	Pat et al. 2020 [23]; Scott et al. 2021 [24]; Gonzalez et al. 2022 [31]; Eriksson et al. 2014 [34]; Flower et al. 2014 [5]

#	Summarised review finding	Methodological limitations	Coherence	Adequacy	Relevance	GRADE-CERQual assessment of confidence	References
		justification and detail on study design and data collection methods, no reflexivity and unclear recruitment and possible selection bias, unclear ethical considerations)	complexity of the data from the primary studies.		relevant to the review question in terms of age of participants included and the potential for inclusion of data related to patients acute UTIs.	No/Very minor concerns regarding adequacy, and Minor concerns regarding relevance	
**HEALTHCARE PROFESSIONALS**							
**8**	There are differences in the use of, and reason for, conducting urine culture.	Minor concerns<br><br>**Explanation:** Minor concerns regarding methodological limitations: two studies of moderate quality and one study of high quality (lack of justification and detail on study design, no reflexivity)	No/Very minor concerns<br><br>**Explanation:** No/ very minor concerns regarding coherence: The review finding reflects the variation and complexity	Serious concerns<br><br>**Explanation:** Serious concerns regarding adequacy: three studies contributing. Two of these offered very thin data.	No/Very minor concerns<br><br>**Explanation:** No/ very minor concerns regarding relevance: three studies that were directly relevant to the review question.	Low confidence<br><br>**Explanation:** Minor concerns regarding methodological limitations, No/Very minor concerns regarding coherence, Serious concerns regarding	Flower et al. 2015 [22]; Cooper et al., 2020 [39]; Larcombe 2012 [38]

#	Summarised review finding	Methodological limitations	Coherence	Adequacy	Relevance	GRADE-CERQual assessment of confidence	References
			of the contributing primary studies.			adequacy, and No/Very minor concerns regarding relevance	
**9**	Making the correct diagnosis is important and HCPs worry about missing serious disease.	Minor concerns<br><br>**Explanation:** Minor concerns regarding methodological limitations: two studies of moderate quality (lack of justification and detail on study design, no reflexivity)	No/Very minor concerns<br><br>**Explanation:** No/ very minor concerns regarding coherence: The review finding reflects the themes identified from the contributing primary studies.	Serious concerns<br><br>**Explanation:** Serious concerns regarding adequacy: two studies contributing of which one of these offered very thin data.	No/Very minor concerns<br><br>**Explanation:** No/ very minor concerns regarding relevance: two studies that were directly relevant to the review question.	Low confidence<br><br>**Explanation:** Minor concerns regarding methodological limitations, No/Very minor concerns regarding coherence, Serious concerns regarding adequacy, and No/Very minor concerns regarding relevance	Flower et al. 2015 [22]; Cooper et al. 2020 [39]

#	Summarised review finding	Methodological limitations	Coherence	Adequacy	Relevance	GRADE-CERQual assessment of confidence	References
**10**	HCPs appreciate that rUTIs have a significant impact on quality-of-life.	Minor concerns<br><br>**Explanation:** Minor concerns regarding methodological limitations: one study of moderate quality (lack of justification and detail on study design, no reflexivity)	No/Very minor concerns<br><br>**Explanation:** No/ very minor concerns regarding coherence: The review finding reflects the themes identified from the single contributing primary study.	Serious concerns<br><br>**Explanation:** Serious concerns regarding adequacy: one study contributing rich data.	No/Very minor concerns<br><br>**Explanation:** No/ very minor concerns regarding relevance: one study that was directly relevant to the review question.	Low confidence<br><br>**Explanation:** Minor concerns regarding methodological limitations, No/Very minor concerns regarding coherence, Serious concerns regarding adequacy, and No/Very minor concerns regarding relevance	Flower et al. 2015 [22]
**11**	HCPs believe self-help measures are important to prevent UTI recurrence	Minor concerns<br><br>**Explanation:** Minor concerns regarding methodological limitations: two	Minor concerns<br><br>**Explanation:** Minor concerns regarding	Serious concerns<br><br>**Explanation:** Serious concerns regarding	No/Very minor concerns<br><br>**Explanation:** No/ very minor	Low confidence<br><br>**Explanation:** Minor concerns regarding	Flower et al. 2015 [22]; Cooper et al. 2020 [39]

#	Summarised review finding	Methodological limitations	Coherence	Adequacy	Relevance	GRADE-CERQual assessment of confidence	References
		studies of moderate quality (lack of justification and detail on study design, no reflexivity)	coherence: the review finding represents the findings from most of the primary studies, but one study mentions the potential limitation of self-care in rUTI (as they have likely already tried this prior to consulting)	adequacy: two studies contributing of which one of these offered very thin data.	concerns regarding relevance: two studies that were directly relevant to the review question.	methodological limitations, Minor concerns regarding coherence, Serious concerns regarding adequacy, and No/Very minor concerns regarding relevance	
**12**	Some HCPs are interested in the use of CAM but have significant concerns about their use and admit a lack of knowledge about them.	Minor concerns<br><br>**Explanation:** Minor concerns regarding methodological limitations: one study of moderate quality	No/Very minor concerns<br><br>**Explanation:** No/ very minor concerns regarding	Serious concerns<br><br>**Explanation:** Serious concerns regarding adequacy: one study	No/Very minor concerns<br><br>**Explanation:** No/ very minor concerns regarding	Low confidence<br><br>**Explanation:** Minor concerns regarding methodological limitations,	Flower et al. 2015 [22]

#	Summarised review finding	Methodological limitations	Coherence	Adequacy	Relevance	GRADE-CERQual assessment of confidence	References
		(lack of justification and detail on study design, no reflexivity)	coherence: The review finding reflects the themes identified from the single contributing primary study.	contributing rich data.	relevance: one study that was directly relevant to the review question.	No/Very minor concerns regarding coherence, Serious concerns regarding adequacy, and No/Very minor concerns regarding relevance	
**13**	HCPs feel antibiotics are effective short term but are concerned about their use.	Minor concerns<br><br>**Explanation:** Minor concerns regarding methodological limitations: two studies of moderate quality (lack of justification and detail on study design, no reflexivity)	No/Very minor concerns<br><br>**Explanation:** No/ very minor concerns regarding coherence: The review finding reflects the themes identified from the	Serious concerns<br><br>**Explanation:** Serious concerns regarding adequacy: two studies contributing of which one of these offered very thin data.	No/Very minor concerns<br><br>**Explanation:** No/ very minor concerns regarding relevance: two studies that were directly relevant to the review question.	Low confidence<br><br>**Explanation:** Minor concerns regarding methodological limitations, No/Very minor concerns regarding coherence, Serious concerns regarding	Flower et al. 2015 [22]; Cooper et al. 2020 [39]

#	Summarised review finding	Methodological limitations	Coherence	Adequacy	Relevance	GRADE-CERQual assessment of confidence	References
			contributing primary studies.			adequacy, and No/Very minor concerns regarding relevance	
**14**	Attitudes towards when to refer to secondary care and its benefits varied amongst HCPs.	Minor concerns<br><br>**Explanation:** Minor concerns regarding methodological limitations: one study of moderate quality and one study of high quality (lack of justification and detail on study design, no reflexivity)	No/Very minor concerns<br><br>**Explanation:** No/ very minor concerns regarding coherence: The review finding reflects the themes identified from the contributing primary studies.	Serious concerns<br><br>**Explanation:** Serious concerns regarding adequacy: two studies contributing very thin data.	No/Very minor concerns<br><br>**Explanation:** No/ very minor concerns regarding relevance: two studies that were directly relevant to the review question.	Low confidence<br><br>**Explanation:** Minor concerns regarding methodological limitations, No/Very minor concerns regarding coherence, Serious concerns regarding adequacy, and No/Very minor concerns regarding relevance	Cooper et al. 2020 [39]; Larcombe 2012 [38]

#	Summarised review finding	Methodological limitations	Coherence	Adequacy	Relevance	GRADE-CERQual assessment of confidence	References
**15**	HCPs must consider and balance several competing interests when managing rUTI.	Minor concerns<br><br>**Explanation:** Minor concerns regarding methodological limitations: one study of high quality and two studies of moderate quality (lack of justification and detail on study design, no reflexivity)	No/Very minor concerns<br><br>**Explanation:** No/ very minor concerns regarding coherence: The review finding reflects the complexity of the themes identified from the contributing primary studies.	Serious concerns<br><br>**Explanation:** Serious concerns regarding adequacy: three studies contributing of which two of these offered very thin data.	No/Very minor concerns<br><br>**Explanation:** No/ very minor concerns regarding relevance: three studies that were directly relevant to the review question.	Low confidence<br><br>**Explanation:** Minor concerns regarding methodological limitations, No/Very minor concerns regarding coherence, Serious concerns regarding adequacy, and No/Very minor concerns regarding relevance	Flower et al. 2015 [22]; Cooper et al. 2020 [39]; Larcombe 2012 [38]
